# A Brain Imaging Study on the Association Between Chess Expertise and Visual Spatial Working Memory in School-Aged Children

**DOI:** 10.3390/brainsci16070734

**Published:** 2026-07-11

**Authors:** Xingjie Hao, Zongchao Chang, Youfa Li

**Affiliations:** 1 Collaborative Innovation Center of Assessment Toward Basic Education Quality, Beijing Normal University, Beijing100875, China; 2 Department of Sports, Beijing Children’s Palace, Beijing 100061, China; 3 College of Physical Education, Minzu University of China, Beijing 100074, China

**Keywords:** chess, school-aged children, visual spatial working memory, brain morphometry, resting-state brain function

## Abstract

**Highlights:**

**What are the main findings?**

- Expert school-aged chess players demonstrate significantly higher accuracy and shorter reaction times in visual spatial working memory (VSWM) tasks compared to novices.
- Multi-modal neuroimaging reveals that childhood chess expertise co-varies with phenotypic variations in gray matter volume, cortical thickness, and distinct resting-state spontaneous BOLD fluctuation indices across frontoparietal and limbic regions.

**What are the implications of the main findings?**

- The tight linear correlation between right subparietal sulcus thickness and VSWM accuracy highlights a specialized neural configuration associated with intensive strategic experience in children.
- These neuroscientific insights underscore the cognitive benefits of mind sports, providing empirical support that aligns with quality education and “double reduction” educational reforms.

**Abstract:**

**Objectives:** To explore the brain imaging correlates of the association between chess skill level and visual spatial working memory (VSWM) in school-aged children. **Methods:** This study analyzed a final sample of 20 school-aged children (aged 10–15 years, mean age of 13.48 ± 1.23 years; including 9 experts and 11 novices) selected from an initial pool of 40 recruited participants from primary and secondary schools and chess clubs in Beijing. They were divided into an experimental group (expert-level school-aged children from a Beijing chess club) and a control group (novice-level children from a Beijing primary and secondary school who had not systematically participated in chess courses but understood basic chess knowledge). The N-back task was used to assess behavioral differences in VSWM. Voxel-based morphometry (VBM) and surface-based morphometry (SBM) techniques were employed to analyze differences in gray matter volume and cortical thickness in the brain structure of expert- and novice-level children. Differences in resting-state functional imaging were also analyzed. **Results:** The results showed the following: (1) The expert-level children demonstrated significantly higher accuracy and shorter reaction times in VSWM tasks compared to novices. (2) Differences in gray matter volume and cortical thickness were observed between the expert and novice groups. (3) Expert-level children showed significantly higher resting-state ALFF/fALFF values in the dorsolateral superior frontal gyrus, cingulate gyrus, and orbitofrontal cortex compared to novices. **Conclusions:** In conclusion, expert-level school-aged children exhibit distinctive regional structural and intrinsic functional profiles. These non-causal neural variations co-vary with selective cognitive baselines characterizing specialized low-load visual spatial processing rather than a generalized working memory expansion, reflecting brain signatures associated with varying tiers of chess expertise.

The mental development of children and adolescents is of paramount importance, impacting national development and the nation’s future. In conjunction with China’s long-standing quality education reforms, families, schools, and society have all invested in non-disciplinary education alongside academic subjects to foster students’ comprehensive literacy [1]. To alleviate the academic burden on primary and secondary school students, catalyze holistic reform in basic education, and construct a new, student-centered educational ecosystem [2], the “double reduction” policy has been continuously deepened, leading to the widespread implementation of non-disciplinary courses both inside and outside of school. Among these, mind sports possess unique educational values and functions, contributing to the implementation of quality education in schools, which has garnered national attention. At the national top-level design, the General Administration of Sport of China, in the “14th Five-Year Plan for Sports Development,” explicitly designates “strengthening the integration of sports and education” as a core chapter [3]. The latest “Government Work Report” in 2025 also re-emphasized the need to “strengthen the popularization of scientific fitness and health interventions among adolescents” [4]. Under this framework, the “Mind Sports on Campus” initiative has been thoroughly implemented nationwide. For instance, local documents such as the “Shenzhen Chess Development Implementation Plan (2022–2025)” explicitly mandate the “comprehensive popularization of chess on campus,” with the ultimate goal of building a “City of Mind Sports” [5]. Concurrently, chess and Chinese chess have become official events in both the 5th National Mind Sports Games (successfully held in 2023) and the 2022 Hangzhou Asian Games. Guided by these government policies and measures, chess, as an intellectual sport, has received widespread attention from both school and family education. It has been introduced into after-school services in the form of clubs, interest classes, and school-based courses within the physical education curriculum, becoming an integral part of school sports education and playing a crucial practical role in nurturing students. Consistently, a large-scale longitudinal tracking from the ABCD study consortium demonstrated that structured sports and strategic training participation in children—regardless of whether it is team- or individual-based—is significantly associated with superior cognitive test profiles and enhanced subcortical gray matter maturation, reinforcing the developmental value of organized non-disciplinary campus courses [6].

Because chess integrates knowledge and skills such as numbers, space, and permutations, the process of playing involves numerous high-level cognitive abilities and can stimulate corresponding brain functions. Given the positive associations between chess expertise, cognitive abilities, and academic performance, it has gradually become a focal point for research on student cognitive promotion and its corresponding brain imaging in school education. Chess expertise is frequently linked to neural signatures indicative of neuroplasticity, and research on chess is progressively deepening [7]. Using brain imaging techniques from a cognitive neuroscience perspective, studies have gradually revealed structural and functional brain differences between adult chess experts and novices [8]. Adult experts with a history of intensive chess practice demonstrate distinctive baseline configurations in their brain structure and function [9,10]. This provides a research basis for investigating the cognitive profiles and brain structural and functional imaging features associated with varying degrees of chess expertise in school-aged children. Although research on chess skill acquisition is relatively abundant, studies specifically targeting school-aged children at different chess proficiency levels are scarce. Existing issues include the need to refine the cognitive ability metrics used in chess research and the relatively slow exploration of brain imaging studies. Therefore, in the context of the new era, to further explore and validate the educational function of chess, it is necessary to focus on school-aged children with different skill levels, both inside and outside of school. The research question must gradually shift toward identifying the specific neural indicators that co-vary with students’ cognitive profiles, exploring whether brain imaging can delineate divergent structural and functional phenotypes that differentiate proficiency cohorts. Based on this, the present study attempts to explore the differences in brain structure and functional imaging between expert- and novice-level school-aged children who have learned chess, thereby enriching the theoretical understanding of localized structural and functional brain signatures characterizing different proficiency cohorts in basic education-stage children and providing practical references for understanding the neural underpinnings of specialized cognitive traits in children and adolescents.

## 1. Research Procedure

### 1.1. Participants

**Figure 1:**
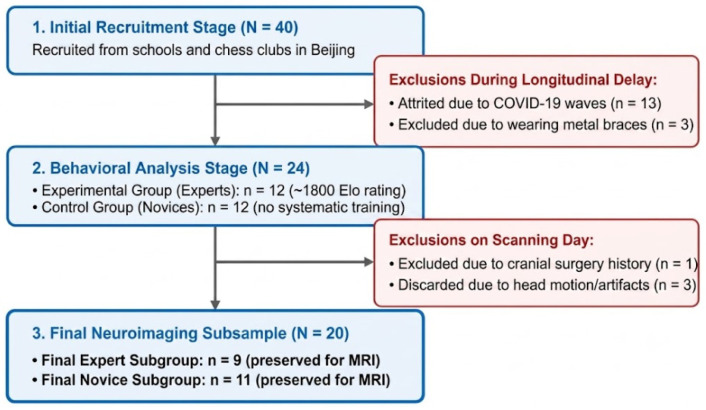
Flowchart of the participant recruitment, exclusion pipeline, and experimental stages.

A total of 40 school-aged children (mean age of 13.48 ± 1.23 years) were initially recruited from primary and secondary schools as well as chess clubs in Beijing. A rigorous filtering pipeline was executed to manage participant recruitment and subsequent data exclusion, ensuring a clear tracking of the sample across experimental phases, as detailed in Figure 1. The specific clinical and behavioral inclusion and exclusion criteria for all participants are comprehensively summarized in Table 1 and Table 2, respectively. Initially, the experiment was scheduled for 26–27 December 2021, but was repeatedly postponed due to the pandemic, and was finally completed between 30 September and 3 October 2022. Due to these consecutive pandemic-related delays, the study suffered a longitudinal attrition of 13 participants who withdrew due to aging crossovers, rating adjustments, or scheduling constraints; an additional 3 participants were excluded due to wearing metal dental braces. Concurrently, 24 matched participants (12 right-handed children in the experimental expert group and 12 right-handed children in the control novice group) successfully completed the behavioral visual spatial working memory (VSWM) N-back testing. The 12 children in the experimental group all held the ‘Class 1 Knight’ title certified by the Chinese Chess Association (benchmarking to approximately 1800 points in the standard international ELO rating system), with a learning history of over 5 years and no significant intra-group differences in chess strength. The 12 children in the control group understood basic chess knowledge but had never systematically participated in chess courses. On the subsequent day of magnetic resonance scanning, 1 control participant was excluded due to a historical profile of cranial surgery, and data from 3 participants (1 expert and 2 novices) were discarded following strict image quality audits due to excessive head motion (maximum translation > 2.0 mm or maximum rotation > 2.0°), incomplete structural sequences, or severe artifacts. Ultimately, a final subset of 20 participants (9 experts in the experimental group, mean age of 13.32 ± 1.27 years; and 11 novices in the control group, mean age of 13.65 ± 1.19 years; age range: 10–15 years) was successfully preserved for the multi-modal neuroimaging (VBM, SBM, ALFF, and fALFF) analyses. The demographic and baseline clinical characteristics of these final MRI participants are systematically compiled in Table 3. All participants and their guardians signed the MRI informed consent form approved by the Institutional Review Board of the Institute of Psychology, Chinese Academy of Sciences (CAS), and received performance-based monetary compensation upon study completion.

### 1.2. Research Methods

#### 1.2.1. Experimental Design

This study employed a cross-sectional design. Participants completed the visual spatial working memory (VSWM) N-back (3 × 3 grid) task test without any intervention. The experiment was divided into a preliminary experiment and a formal experiment; the preliminary experiment served to familiarize participants with the operation tasks and experimental procedures. The formal experiment included 4 blocks, each with 24 trials, for a total of 96 trials. The behavioral evaluation metrics for the VSWM task were accuracy and reaction time. This study utilized chess expertise proficiency as the grouping factor to examine cross-sectional variations in regional gray matter volume, cortical thickness, and resting-state functional indices across distinct proficiency tiers.

#### 1.2.2. Experimental Materials

##### Visual Spatial Working Memory Test

The task was programmed using E-Prime 2.0 (Psychology Software Tools, Sharpsburg, PA, USA). A gray-background square, 9 cm × 9 cm, was divided into a 3 cm × 3 cm black grid, with each small square being 3 cm × 3 cm. A black “X” symbol appeared randomly in one of the nine squares. A “+” sign was displayed in the center of the computer screen as a fixation point for 350 ms. Participants observed the stimulus image, which lasted for 500 ms, followed by a blank screen delay of 1000–1500 ms before the next stimulus image. The 0-back task required participants to remember the position of the first target and observe whether each subsequent stimulus was in the same position. The 1-back task required no response to the first stimulus; thereafter, participants compared the position of each stimulus with the immediately preceding one to observe if their positions were identical. Each experiment lasted 20 min.

##### Magnetic Resonance Imaging Data Acquisition

All participant MRI scans were completed at the MRI Research Center, Institute of Psychology, CAS, using a GE Discovery MR750 3T research-type magnetic resonance imaging scanner (GE Healthcare, Chicago, IL, USA). The scanning sequences included high-resolution T1-weighted structural imaging, resting-state functional magnetic resonance imaging, and the VSWM task. During the scan, participants lay supine in the scanner, their heads stabilized, breathing calmly, and maintaining their most comfortable state to minimize head movement. T1-weighted structural imaging was acquired using a sagittal 3D magnetization-prepared rapid gradient-echo (MPRAGE) sequence, with the following parameters: sagittal plane, 192 slices, repetition time (TR) = 3200 ms, echo time (TE) = 3.44 ms, slice thickness = 1 mm, flip angle = 90°, field of view (FOV) = 256 × 256 mm^2^, and acquisition matrix = 256 × 256. Resting-state functional magnetic resonance imaging (fMRI) data were acquired using an echo-planar imaging (EPI) sequence with the following parameters: axial 33 slices, TE = 30 ms, TR = 3200 ms, flip angle = 90°, slice thickness = 3.5 mm, data matrix = 64 × 64, and FOV = 224 × 224 mm^2^, resulting in an isotropic voxel size of 3.5 × 3.5 × 3.5 mm^3^. A total of 124 functional volumes (timepoints) were acquired for each participant during the 6min and 38s resting-state session. Structural images were acquired to provide anatomical reference for the preprocessing of resting-state MRI data. During the resting-state scan, participants were instructed to remain awake, think about nothing in particular, keep their eyes open to look at the screen image, and keep their heads as still as possible.

### 1.3. Experimental Procedure

**Figure 2:**
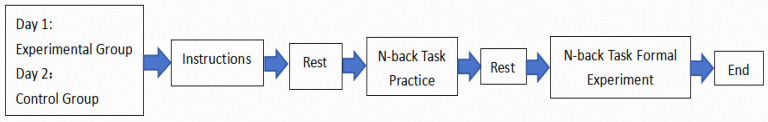
Experimental flowchart.

This experiment used a within-subject design, employing the classic N-back 3 × 3 grid paradigm to examine participants’ visual spatial working memory. The experimental process mainly consisted of testing and scanning parts. Participants first underwent training and testing on the VSWM task, followed by functional magnetic resonance scanning. Considering that school-aged participants might experience discomfort in the experimental environment, a preliminary mock scan was arranged in advance. This allowed participants to be monitored for head motion information and receive feedback in a pediatric MRI simulator to familiarize them with the experimental procedure. Before the scan, participants practiced the VSWM task until they were familiar with it, after which the formal experiment began (Figure 2). Each participant changed clothes before entering the MRI scanner for the scan to reduce potential practice effects, undergoing brain structural and resting-state functional imaging. Before the experiment, participants were required to change clothes and pass through a body metal detector. They then entered the scanner room, lay supine on the scanner bed, wore foam earplugs to reduce instrument noise, and had their heads stabilized with foam pads. They were instructed to remain awake and breathe steadily. The experimenter inquired and then began the experiment. The resting-state scan lasted 6 min and 38 s, and the structural scan lasted 7 min and 35 s.

### 1.4. Magnetic Resonance Imaging Data Analysis and Processing

Preprocessing of high-resolution T1WI and extraction of the cortical surface were performed using automated procedures in the Computational Anatomy Toolbox (CAT12, version 12.8.2), running on the MATLAB 2021a platform (MathWorks, Natick, MA, USA) with Statistical Parametric Mapping (SPM12, version v7771). All structural T1WI images were spatially normalized to the Montreal Neurological Institute (MNI) standard space (specifically the MNI152 template). As per CAT12 analysis, the gray matter (GM) volume and whole-brain cortical thickness for each individual were obtained. This procedure uses tissue segmentation to estimate WM distance and projects the local cortical thickness maximum to other GM voxels using the neighborhood relationship described by the WM distance.

**Figure 3:**
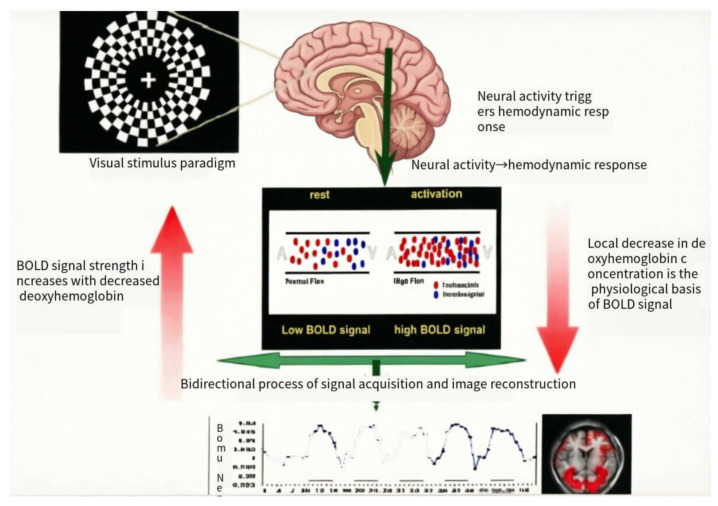
Data processing flowchart.

Resting-state data processing was implemented using MATLAB 2021a, SPM12, and DPABI [11]. Preprocessing of functional imaging data was performed using the DPABISF toolbox based on the MATLAB software platform. To ensure high data quality and eliminate potential noise artifacts, the standardized preprocessing pipeline was executed as follows: initially, the first 10 time points of each functional session were discarded to allow for magnetization stabilization and participant adaptation. Slice-timing correction was then applied to account for temporal acquisition differences between slices within each TR. Functional images were subsequently realigned to the first volume to estimate and correct for head motion. Strict quality control thresholds for head motion were implemented: participants were excluded if their head movement exceeded 2.0 mm of maximum translation or 2.0° of maximum rotation in any directional axis throughout the scan, or if their mean framewise displacement (FD) calculated via Jenkinson’s formula exceeded 0.2 mm. Following realignment, the functional images were coregistered to each participant’s high-resolution T1-weighted structural image, spatially normalized to the standard Montreal Neurological Institute (MNI) space [12], resampled into an isotropic functional voxel size of 3.5 × 3.5 × 3.5 mm^3^, and smoothed with a 6mm Full Width at Half Maximum (FWHM) Gaussian kernel. To further control for physiological noise and motion artifacts, nuisance covariates were regressed out, including the Friston 24-head-motion parameters, global signal, white matter signal, and cerebrospinal fluid (CSF) signal. Finally, a fast Fourier transform was used to convert the dynamic functional matrix into the frequency domain, and a temporal band-pass filter (0.01–0.1 Hz) was applied to reduce low-frequency drift and high-frequency respiratory/cardiac noise, after which Amplitude of Low-Frequency Fluctuation (ALFF) and Fractional Amplitude of Low-Frequency Fluctuation (fALFF) values were quantified and calculated (Figure 3).

(1) ALFF analysis: Amplitude of Low-Frequency Fluctuations (ALFF) was calculated to measure the absolute intensity of spontaneous blood-oxygen-level-dependent (BOLD) signal fluctuations within the low-frequency range (0.01–0.1 Hz). Following standard preprocessing, the time series of each voxel was transformed into the frequency domain using a fast Fourier transform (FFT). The power spectrum was obtained, and the square root was calculated at each frequency across the 0.01–0.1 Hz spectrum. The averaged square root was then designated as the ALFF value, reflecting the absolute magnitude of regional spontaneous neural oscillations.

(2) fALFF analysis: Fractional Amplitude of Low-Frequency Fluctuations (fALFF) represents the proportion of low-frequency activity relative to the total detectable frequency spectrum. It was computed as the ratio of the sum of amplitudes across the low-frequency band (0.01–0.1 Hz) to the sum of amplitudes across the entire detectable frequency range (0–0.25 Hz, determined by the Nyquist frequency based on TR = 3.2 s). By normalizing the BOLD signal across the entire spectrum, fALFF effectively suppresses non-neural physiological background noise—such as cerebrospinal fluid fluctuations, respiratory rhythms, and cardiac cycles—thereby significantly enhancing the spatial sensitivity and specificity of detecting baseline neural signaling profiles.

### 1.5. Statistical Analysis

Independent-sample *t*-tests were performed on the structural and cortical surface metrics using SPM12/CAT12 packages. To establish rigorous confirmatory neural differences between cohorts, whole-brain morphometric thresholds were constrained to a voxel-level *p* < 0.001 with a False Discovery Rate (FDR)-corrected cluster-level *p* < 0.05. For subsequent regional analysis, post hoc independent-sample *t*-tests were conducted within SPSS 22.0 using the extracted parameters from these corrected regions, with *p* < 0.05 defining significance. Crucially, post hoc Pearson correlation analyses exploring relationships among extracted gray matter volume, cortical thickness, functional signaling indices, and behavioral VSWM scores were treated as exploratory regional investigations, thresholded at an uncorrected *p* < 0.05 due to the small-sample character of the neuroimaging sub-cohort. Resting-state fMRI data were preprocessed using DPABI v6.1 software in the MATLAB environment. Whole-brain level independent-sample *t*-tests were performed on the calculated ALFF and fALFF values, with a voxel-level *p* < 0.001, a cluster-level *p* < 0.01, and a voxel count > 100 considered significantly different (two-tailed). These statistical boundaries delineated the baseline variations in spontaneous BOLD fluctuation indices between proficiency cohorts. To explore whether variations in resting-state intrinsic activity associated with chess expertise co-varied with the VSWM task, correlation analyses were performed between the behavioral difference results from the N-back task and the extracted differential functional signal regions between the two groups.

## 2. Research Results

### 2.1. Differences in Visual Spatial Working Memory Between Expert- and Novice-Level School-Aged Children

To maximize statistical power for behavioral profiling, behavioral performance data from the VSWM task were processed based on the full initially matched behavioral sample (N = 24; 12 experts and 12 novices), as detailed in Table 4. Independent-sample *t*-tests were executed to analyze behavioral differences, with group tier (experimental group: expert level; control group: novice level) serving as the between-subject variable. The descriptive and inferential statistics for VSWM accuracy and reaction times are summarized in Table 5 and Table 6, respectively.

For 0-back accuracy, the independent-sample *t*-test revealed that the experimental group’s performance was significantly higher than that of the control group (t(22) = 4.971, *p* < 0.001, Cohen’s d = 2.081). However, for 1-back accuracy, no significant difference was observed between the two cohorts (t(22) = 0.154, *p* = 0.879, Cohen’s d = 0.131). This pattern reflects a statistically significant difference in tracking visual spatial orientations between the expert and control cohorts exclusively under baseline working memory loads (0-back). The absence of significant group differences in the higher-load 1-back task suggests that the captured behavioral edge is workload-dependent rather than representing a broad VSWM enhancement.

Regarding reaction time (RT), the independent-sample *t*-test demonstrated that the experimental group exhibited a significantly shorter (faster) RT in the 0-back task compared to the control group (t(22) = −2.438, *p* = 0.023, Cohen’s d = −0.992). For the 1-back task, the difference in RT between the two groups did not reach statistical significance (t(22) = −1.190, *p* = 0.246, Cohen’s d = −0.492).

It is worth noting that although the behavioral evaluation utilized the full sample of 24 participants to maintain behavioral robustness, the subsequent multi-modal neuroimaging analyses were restricted to a refined subset of 20 participants (9 experts and 11 novices) due to strict scan-day movement and metal exclusion criteria.

### 2.2. Differences in Brain Gray Matter Volume Between Expert- and Novice-Level School-Aged Children

Structural MRI data, specifically brain gray matter volume (GMV), were computed and analyzed using voxel-based morphometry (VBM) to compare the GMV differences between expert- and novice-level school-aged children, thereby analyzing the structural neural characteristics associated with intensive chess experience on GMV in this population.

**Figure 4:**
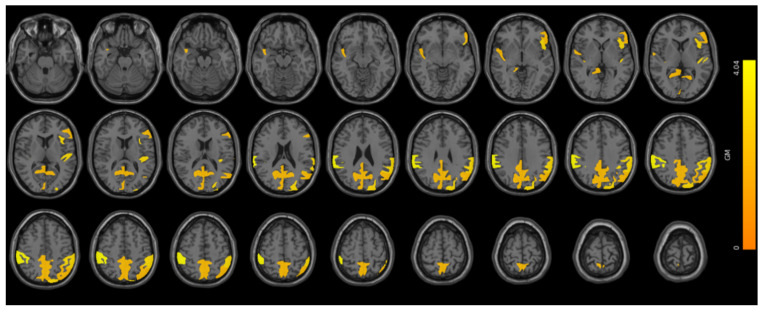
Differences in gray matter volume between the experimental and control groups.

Voxel-based morphometry (VBM) was employed to evaluate group differences in gray matter volume (GMV). As summarized in Table 7, the experimental group showed significantly greater GMV relative to the control group across 12 brain regions (all *p* < 0.05, independent-sample *t*-test). Specifically, the regions with larger GMV in the experimental group included the left superior occipital gyrus (L SOG), bilateral precuneus (PCu), bilateral supramarginal gyrus (SMG), left frontal operculum (L FO), right posterior cingulate cortex (R PCC), right cuneus, right planum polare (R PP), left angular gyrus (L ANG), left transverse temporal gyrus (L TTG), and left triangular part of the inferior frontal gyrus (L IFGtriang), as systematically visualized in Figure 4.

Anatomically, the identified regions span multiple distinct brain systems: the L FO and L IFGtriang reside in the frontal lobe; the bilateral PCu, bilateral SMG, and L ANG are situated in the parietal lobe; the L SOG and right cuneus represent subregions of the occipital lobe; the R PP and L TTG belong to the temporal lobe; and the R PCC constitutes a core structure of the limbic system. Taken together, these observations indicate that expert-level proficiency in school-aged children is associated with greater regional GMV across subregions of the frontal, parietal, occipital, and temporal lobes, as well as within limbic system structures.

The VBM comparison of gray matter volume, as shown in Table 8, revealed no significant differences between the experimental and control groups in the gray matter volume of the left superior frontal gyrus (L SFG), left middle frontal gyrus (L MFG), left postcentral gyrus (L poCG), right postcentral gyrus (R poCG), left superior parietal gyrus (L SPG), right superior parietal gyrus (R SPG), right angular gyrus (R ANG), and right superior occipital gyrus (R SOG). These findings indicate that the differences in brain gray matter volume associated with chess expertise in school-aged children exhibit regional selectivity within the frontoparietal networks, with several anticipated cortical regions showing no statistically significant variations under the current age and developmental baseline.

### 2.3. Differences in Brain Cortical Thickness Between Expert- and Novice-Level School-Aged Children

Surface-based morphometry (SBM) was used to compute and analyze the cortical thickness of the brain structure, comparing differences between expert- and novice-level school-aged children to characterize cortical thickness phenotypes associated with chess expertise.

**Figure 5:**
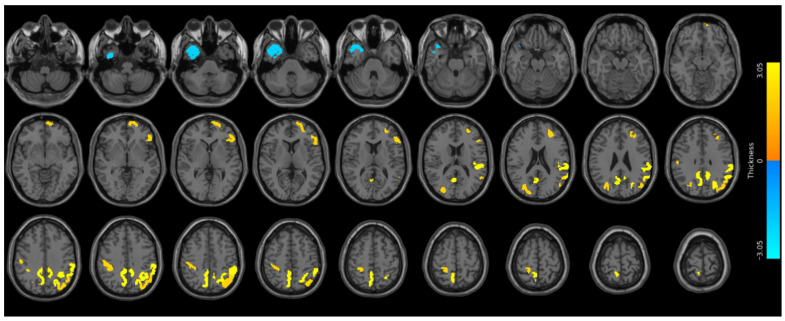
Differences in cortical thickness between the experimental and control groups.

Surface-based morphometry (SBM) comparisons revealed a selective cross-sectional divergence in cortical thickness between the expert and novice cohorts (Table 9). Relative to the novice control group, expert-level school-aged children demonstrated significantly higher cortical thickness baseline profiles across 12 distinct anatomical regions (all *p* < 0.05, independent-sample *t*-test), which span the frontal lobe (the left triangular part of the inferior frontal gyrus [IFGtriang], left middle frontal sulcus [mfs], and left transverse frontopolar gyri and sulcus [tFPG/s]), the parietal lobe (the right precuneus [PCu], left angular gyrus [ANG], left supramarginal gyrus [SMG], left sulcus intermedius primus of Jensen [si_Jensen], left interparietal and transverse parietal sulcus [IPS/tPS], right postcentral sulcus [poCS], and bilateral subparietal sulcus [sps]), and the occipital lobe (the right superior occipital and transverse occipital sulcus [SOS/tOS]), as comprehensively illustrated in Figure 5. Conversely, a distinctive anatomical variation was captured in the temporal structures, wherein the expert group exhibited a significantly thinner cortical baseline profile selectively within the right temporal pole (TP) compared to novices (t = −2.34, *p* = 0.030). Taken together, these SBM observations demonstrate that varying tiers of chess expertise co-vary with distinctive localized cortical thickness phenotypes across frontoparietal, occipital, and anterior temporal structures in school-aged children.

The SBM comparison of cortical thickness, as shown in Table 10, revealed no significant differences between the experimental and control groups in the cortical thickness of the left superior frontal gyrus (L SFG), left middle frontal gyrus (L MFG), right supramarginal gyrus (R SMG), left postcentral gyrus (L poCG), right postcentral gyrus (R poCG), left superior parietal gyrus (L SPG), right superior parietal gyrus (R SPG), right angular gyrus (R ANG), and right superior occipital gyrus (R SOG). This may be attributable to age and brain structural development.

### 2.4. Differences in Resting-State Spontaneous Functional Signaling Between Expert- and Novice-Level School-Aged Children

In this study, the analysis of low-frequency amplitudes primarily included the computation and analysis of whole-brain voxel-wise ALFF and fALFF values.

#### 2.4.1. ALFF Differences Between Expert- and Novice-Level School-Aged Children

Resting-state fMRI data from expert- and novice-level school-aged children were processed to analyze ALFF differences, clarifying the functional baseline profiles corresponding to varying chess proficiency tiers in this population.

**Figure 6:**
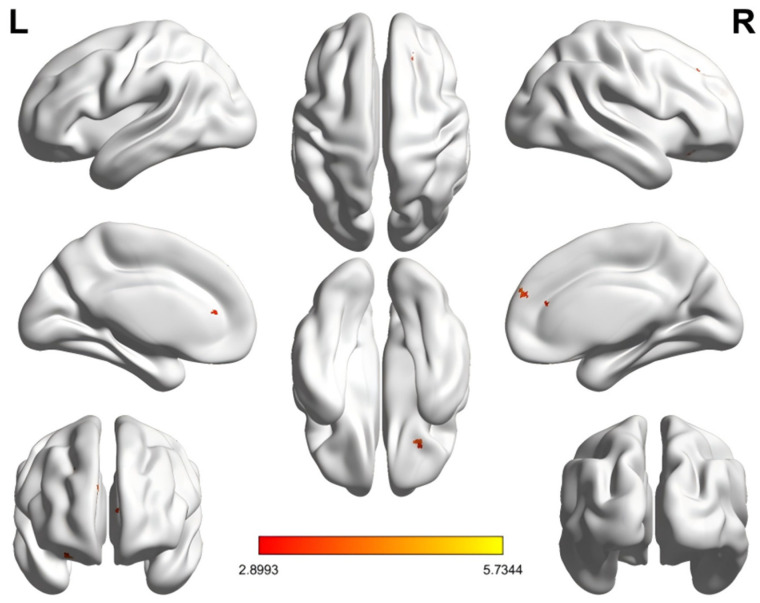
Differences in ALFF values between the experimental and control groups. Note: The ALFF value in the right dorsolateral superior frontal gyrus (superior frontal gyrus, dorsolateral) of the experimental group was significantly higher than that of the control group. The peak coordinates of this cluster were (33, 42, −6), and the voxel size was 198.

Analysis of ALFF values between expert- and novice-level school-aged children revealed that the ALFF value in the dorsolateral superior frontal gyrus (Figure 6) was significantly higher in the experimental group than in the control group. This demonstrates a localized difference in baseline spontaneous low-frequency fluctuations within this specific region between the two groups.

#### 2.4.2. fALFF Differences Between Expert- and Novice-Level School-Aged Children

Resting-state fMRI data from expert- and novice-level school-aged children were processed to analyze fALFF differences, further delineating the intrinsic functional signatures associated with varying chess expertise tiers in school-aged children.

**Figure 7:**
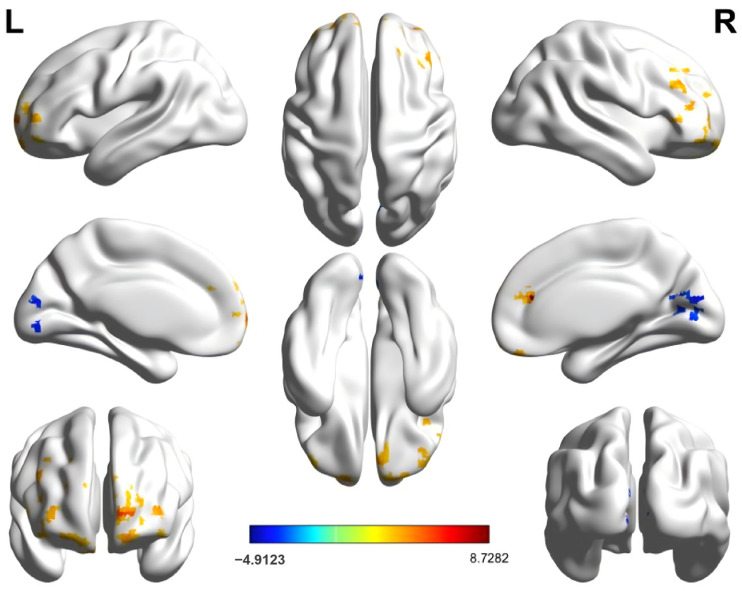
Differences in fALFF values between the experimental and control groups.

Analysis of fALFF values between expert- and novice-level school-aged children revealed that the fALFF values in the anterior cingulate cortex (ACC), dorsolateral superior frontal gyrus (SFGdl), and orbitofrontal cortex (OFCant) were significantly higher in the experimental group than in the control group (Table 11). Conversely, the fALFF value in the calcarine cortex (CAL) was significantly lower in the experimental group (Figure 7). Anatomically, the bilateral SFGdl and OFCant are situated within the frontal lobe, the ACC constitutes a component of the limbic system, and the CAL represent a subregion of the occipital lobe. In summary, the localized fALFF variations demonstrates that the expert cohort exhibits higher intrinsic functional signaling indices across selective frontal and limbic structures, alongside lower intrinsic functional indices within selective occipital regions, compared to the novice control cohort.

### 2.5. Correlation Analysis Between Brain Cortical Thickness and Visual Spatial Working Memory

The CAT12 software was used to extract regional brain cortical thickness profiles in expert- and novice-level school-aged children. To actively explore structural behavioral trends under small-sample constraints, post hoc exploratory Pearson correlation analyses were executed to examine the relationship between localized cortical thickness values and VSWM performance metrics.

**Figure 8:**
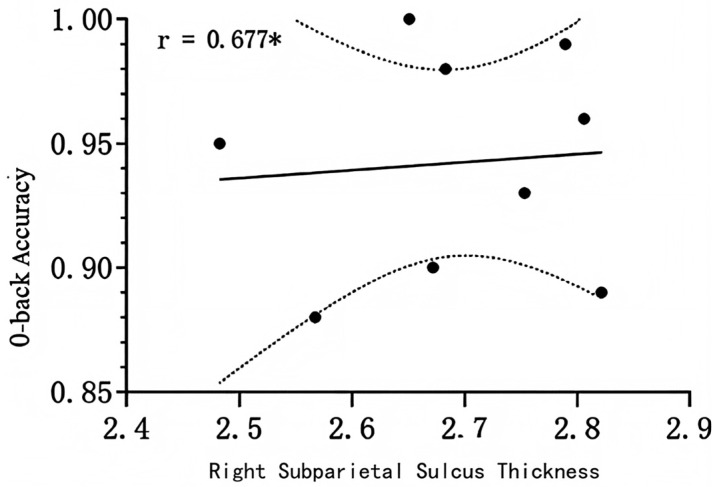
Correlation between right subparietal sulcus thickness and visual spatial working memory performance (quantified by 0-back task accuracy) in the experimental group. The solid line represents the expert group, and the dashed line represents the novice group. Black dots indicate individual participant scores. * *p* < 0.05.

**Figure 9:**
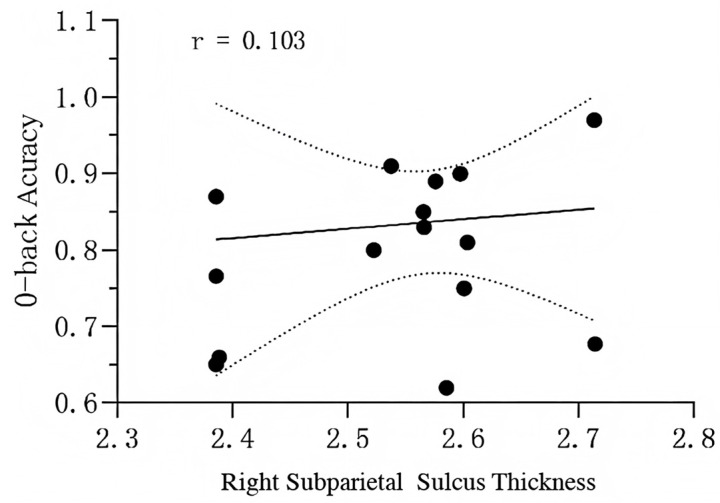
Correlation between right subparietal sulcus thickness and visual spatial working memory performance (quantified by 0-back task accuracy) in the control group. The solid line represents the expert group, and the dashed line represents the novice group. Black dots indicate individual participant scores.

The statistical analysis revealed a significant positive correlation between the cortical thickness values in the right subparietal sulcus and 0-back task accuracy in the experimental group (r = 0.677, *p* < 0.05) (Figure 8). Conversely, no statistically significant correlation was observed within the control group (Figure 9).

## 3. Analysis and Discussion

### 3.1. Comparative Analysis of Brain Gray Matter Divergence Across Chess-Playing School-Aged Children

In this study, we explored the structural neural phenotypes differentiating proficiency cohorts in terms of gray matter volume (GMV) in school-aged children, elucidating the exploratory covariance between regional GMV features and chess proficiency tiers. VBM was used to analyze structural MRI data, specifically brain GMV, comparing differences between expert- and novice-level children. Our results found that expert-level children had significantly larger GMV than novices in the left angular gyrus, right precuneus, left precuneus, left superior occipital gyrus, right cuneus, left frontal operculum, right posterior cingulate, right planum polare, left supramarginal gyrus, right supramarginal gyrus, left triangular part of the inferior frontal gyrus, and left transverse temporal gyrus. These regions belong to the frontal, parietal, occipital, and temporal lobes. Based on functional brain research, studies examining structural changes have found that chess experts, compared to amateurs, exhibit significantly smaller GMV in the bilateral caudate nucleus [13]. Prior evidence suggests that the cognitive expertise of chess masters largely depends on rapid, automatic processing [14,15,16,17,18], a capacity thought to be mediated by the caudate nucleus, which may also be susceptible to age-related GMV reduction [19]. Our study indicates, combined with the behavioral results, that the strengthening of the visual-stimulus visual association in expert-level school-aged children, which demonstrates widespread visual attention network involvement, leads to heightened intrinsic baseline profiles in related brain regions [20]. This suggests that chess expertise is associated with a higher phenotypic baseline in GMV within these regions, which co-varies with specific cognitive profiles consistent with an inverted U-shaped development pattern [13]. This finding aligns with Hu’s (2011) results regarding the impact of abacus-based mental calculation on children’s brain development [21]. Varying levels of chess expertise exhibit a strong statistical coupling with divergent functional and structural brain signatures, aligning with a cross-sectional structural functional bifurcation between proficiency cohorts [13,16]. The evidence found in this study confirms that distinct individual variations in chess proficiency cross-sectionally co-vary with regional GMV metrics within parts of the frontal, parietal, occipital, and temporal lobes, as well as the limbic system, reflecting convergent phenotypic benchmarks rather than training-induced alterations.

### 3.2. Analysis of Brain Cortical Thickness Changes in Chess-Playing School-Aged Children

The CAT12 comparative analysis of cortical thickness revealed that expert-level school-aged children had significantly thicker cortex than novices in the left inferior frontal gyrus triangular part, left angular gyrus, left supramarginal gyrus, right precuneus, middle frontal sulcus, transverse frontopolar gyri and sulcus, sulcus intermedius primus of Jensen, interparietal and transverse parietal sulcus, superior occipital and transversal sulcus, postcentral sulcus, left subparietal sulcus, and right subparietal sulcus. Conversely, the right temporal pole’s cortical thickness was significantly thinner. Further Pearson correlation analysis revealed a significant positive correlation between the change in cortical thickness of the right subparietal sulcus and the improvement in 0-back accuracy in VSWM among expert-level children. As chess involves complex and variable skills, and playing requires the mobilization of multiple high-level cognitive abilities [22], the achievements of chess experts are primarily attributed to years of skill practice and cognitive ability. Voxel-based measurements have found that adult experts, compared to adult novices, show significant reductions in cortical thickness in the prefrontal cortex [23], parietal gyri, occipitotemporal junction, and bilateral frontoparietal regions. These observations suggest that divergent cortical thickness profiles between proficiency cohorts are cross-sectionally associated with specialized cognitive traits involving reasoning, rather than reflecting prospective structural changes over time. Consistently, a recent systematic review confirmed that expert chess players exhibit enhanced functional connectivity within networks underlying cognitive control and decision making—such as the dorsolateral prefrontal cortex (dlPFC) and the ACC—alongside reduced gray matter volume in the occipito-temporal junction, collectively reflecting an increased neural efficiency associated with expertise [6]. Partially mirroring these localized structural reductions, a landmark surface-based study on Chinese chess experts by Ouellette et al. (2020) further demonstrated that adult masters exhibit widespread cortical thinning due to adaptive neural pruning, while these structurally refined regions paradoxically display heightened global functional connectivity to support expert-level performance [24].

Moreover, previous research has also noted that varying chess expertise co-varies with distinctive baseline phenotypes of cortical complexity in regions involved in visual spatial information processing tasks. The cortex of chess experts displays specialized patterns during cognitive tasks, where higher proficiency aligns with distinctive structural and functional brain signatures. This demonstrates that distinctive structural and functional profiles are sustained within the parietal and frontal lobes of expert-level school-aged children, features that closely mirror superior profiles in complex cognitive behaviors. Research on cortical thickness has shown that professional chess players have cortical thinning in the precuneus, and this thinning is correlated with chess skill level and training duration [25]. The distinct cortical phenotypes in chess experts may reflect baseline variations in neural efficiency and pruning templates during adolescence [26]. Based on this evidence, we examined cortical changes in expert-level school-aged children. We found that the cortical thickness in parietal regions related to visual spatial abilities was significantly thicker in experts than in novices, and it showed a significant positive correlation with brain regions related to visual spatial working memory. This suggests a distinct phenotypic divergence where the cortical thickness of these brain regions exhibits a specialized baseline linked to individual chess expertise. The thickening of the cortex in the interparietal sulcus (part of the parietal lobe) indicates enhanced visual spatial information processing ability and is related to mathematical geometry [24]. Therefore, the cortical thickness variations in the brain regions of expert-level school-aged children may reflect a specialized neural configuration associated with superior VSWM performance.

### 3.3. Comparative Analysis of Intrinsic Functional Signaling Variations in Chess-Playing School-Aged Children

In this study, we explored the variations in low-frequency amplitudes associated with chess expertise in school-aged children, demonstrating that these intrinsic functional signatures exhibit a pronounced divergence linked to expertise levels. This also confirms that chess expertise is closely associated with phenotypic variations in the visual spatial abilities of school-aged children. The ALFF and fALFF analyses revealed that children learning chess showed higher baseline spontaneous signal intensity in the dorsolateral superior frontal gyrus, cingulate gyrus, and orbitofrontal cortex. Behavioral data analysis showed that expert-level children were significantly better than novices in 0-back accuracy and reaction time. This study confirms that differences in resting-state BOLD fluctuation indices exist between expert- and novice-level school-aged children. The regions demonstrating divergent functional signaling indices belong to the frontal lobe, limbic system, and occipital lobe. This functional brain difference occurs not only in adult professional players but also in high-level school-aged players as their skill level, training time, and frequency increase. Consistently, a recent whole-brain study by Song et al. (2022) demonstrated that professional chess players exhibit significantly increased functional connectivity homogeneity within the ACC, confirming its critical role in expertise-related cognitive networks [27].

Furthermore, expert-level children exhibited prominent baseline signals across more brain regions than novices, and these regions overlapped with those typically engaged during the VSWM task. This finding of resting-state ALFF/fALFF variations is supported by the results of Hänggi et al. (2014), who confirmed increased spontaneous functional activity in the frontal and parietal cortical regions of adult chess experts [23]. Other studies are also consistent with our results. Krawczyk et al. (2011) investigated the neural correlates of visual recognition in chess experts and novices and found activation in the posterior cingulate, orbitofrontal cortex, and right temporal cortex in experts [28], which aligns with our conclusions. The ability of chess experts to recognize board layouts and piece combinations is significantly stronger than that of novices, consistent with previous research on the recognition advantages of chess experts [28]. The heightened low-frequency fluctuation of local brain regions in chess-playing children suggests that their chess ability may be related to their visual perspective-switching ability, allowing them to effectively recognize shapes and scene stimuli. This result is consistent with that of Song et al. (2020), who found that chess practice is positively associated with phenotypic variations in brain regions associated with cognitive abilities in professional players [10]. Foster (2000) found that chess experts activate working memory patterns related to geometric shapes, revealing functional recruitment from the prefrontal to the temporal lobes [29]. Pereira et al. (2020) found significant functional engagement in the prefrontal cortex in both adult and adolescent groups, which was positively correlated with task difficulty—the higher the difficulty, the stronger the BOLD response [30]. However, the functional profiles differed between the two groups: adults showed greater prefrontal recruitment during complex tasks, whereas adolescents showed greater prefrontal involvement during medium-difficulty tasks. This result confirms that brain activity patterns are influenced by chess expertise or skill level and also demonstrates the multi-regional, coordinated brain activation pattern required when players play chess. Our study, based on group comparisons of resting-state function, showed that expert-level school-aged children have greater spontaneous BOLD fluctuation profiles in the dorsolateral superior frontal gyrus, cingulate gyrus, and orbitofrontal cortex.

### 3.4. Exploratory Correlation Analysis Between Cortical Thickness and Visual Spatial Working Memory in Chess-Playing School-Aged Children

By executing an exploratory correlation analysis between regional cortical thickness profiles and VSWM performance metrics within the expert-level school-aged children, a significant positive correlation was uncovered between right subparietal sulcus thickness and 0-back accuracy in VSWM (r = 0.677, *p* < 0.05). This provides preliminary, exploratory evidence regarding structural behavioral coupling related to VSWM in expert children, demonstrating that distinct structural properties in brain regions associated with VSWM exhibit a pronounced variance linked to individual chess expertise. Further research has confirmed a correlation between VSWM levels and geometry performance in middle school students. Chess skill relies on visual spatial ability to locate information on the board and pieces, and VSWM is related to spatial orientation ability [31]. Chess skills may be related to spatial imagination in mathematical ability [32]. A study on the N-back VSWM task found that the brain regions involved in the task—primarily the bilateral dorsolateral prefrontal cortex, bilateral ventrolateral prefrontal cortex, dorsal cingulate gyrus, orbitofrontal cortex, pericalcarine cortex, left fusiform gyrus, right medial cingulate gyrus, superior occipital gyrus, precentral gyrus, and left dorsolateral superior frontal gyrus—showed enhanced task-induced activation [26]. These findings are consistent with the conclusions of our study, confirming that chess skill level co-varies with the intrinsic functional properties of brain regions related to VSWM in school-aged children. The subparietal sulcus belongs to the parietal lobe, which is mainly related to sensation and cognition, responsible for spatial relationships, and capable of analyzing and comparing the moving positions of objects. The parietal lobe plays a dominant role in processing short-term spatial memory information [33,34]. Brain regions for number recognition and spatial processing overlap in the parietal lobe [35]. Therefore, these data demonstrate a distinct structural behavioral coupling within the expert cohort, where individuals with higher cortical thickness profiles in specific parietal regions exhibit systematically higher 0-back tracking accuracy. However, the lack of significant variance under the 1-back condition emphasizes that these cross-sectional data cannot support claims regarding broad cognitive transfer to academic mathematical logical competencies or sweeping educational benefits.

## 4. Limitations and Future Directions

Despite the boundaries of the research design, a comprehensive evaluation of the study’s framework requires a balanced consideration of its core findings and its methodological scope. Distinctively, a fundamental strength of this study lies in its success in establishing robust, multi-modal neural and behavioral correlations associated with chess expertise in school-aged children. By integrating VBM, SBM, and the resting-state functional metrics ALFF and fALFF, this work provides a valuable cross-sectional mapping that highlights how specialized visual spatial training correlates with specific localized structural functional variations in the developing brain. These demonstrated correlations offer a timely and solid neuroscientific reference that aligns with and supports recent school quality education and “double reduction” educational reforms, confirming that systematic mind-sport engagement is strongly associated with enhanced processing skills in students.

Nevertheless, several critical limitations and potential sources of bias must be acknowledged, particularly regarding the interpretation of these correlations: First, the final sample size utilized for neuroimaging analysis (n = 20; 9 experts and 11 novices) is relatively small, which inherently limits overall statistical power and precludes high-dimensional multiple comparison corrections or path-analysis modeling across all modalities. This constraint was heavily exacerbated by the overlapping waves of the COVID-19 pandemic. Due to strict quarantine alignments, scheduling conflicts, and age cross-overs during the sequential postponements and final strict containment measures between late 2021 and 2022, the study suffered an unavoidable longitudinal attrition of 13 participants. This reduction in the final participant cohort restricts the generalizability of the localized neural variations. Second, the existing neuroimaging literature specifically targeting developing school-aged children across distinct chess proficiency tiers remains exceptionally scarce. This relative scarcity of historical scientific data limits our capacity to benchmark these localized, cross-sectional frontoparietal and limbic variations against large-scale, long-term standardized normative reference models. Third, while the identified neural correlations represent a major strength of this study, the cross-sectional rather than longitudinal design means our findings are strictly correlational rather than causal. We cannot definitively establish whether intensive chess expertise directly drives these neuroplastic changes, or whether baseline intelligence quotient (IQ) and preexisting neurodevelopmental predispositions naturally inclined certain children to excel in both chess and visual spatial tasks. Crucially, the current design could not strictly control for potential Socio-Economic Status (SES) biases. Families residing in urban areas who systematically invest in long-term elite chess club memberships might systematically covary with higher household income, unique educational resources, or specific parental nurturing paradigms, which may independently incline these developing children toward superior cognitive baselines. Fourth, regarding the behavioral classification framework, the study lacked continuous long-term dynamic monitoring of absolute ranking adjustments. Moreover, given the contemporary proliferation of online chess platforms, the daily casual chess exposure within the novice control group was not strictly logged via long-term digital auditing, which represents a potential baseline confound. Finally, from a methodological perspective, TR = 3200 ms parameterized during resting-state fMRI acquisition was relatively large, which lowers the temporal resolution and potentially induces temporal aliasing of higher-frequency physiological noise (such as cardiac and respiratory cycles) into the lower-frequency fluctuations characteristic of resting-state BOLD signals.

To bridge the gap from these established correlations to causality, the immediate next steps and future perspectives require shifting from purely resting-state observations to specialized chess-chunking task-fMRI paradigms to resolve real-time dynamic network reconfigurations under cognitive load. Methodologically, future replications should utilize accelerated echo-planar imaging sequence clusters (e.g., multiband acceleration with a shorter TR less than 2000 ms) to further resolve fine-grained dynamic functional connectivity. Most importantly, future investigations must prioritize larger, multi-center sample cohorts and prospective longitudinal intervention designs. Tracking entirely naïve school-aged cohorts over a systematic mind-sport training period (such as a 12-week intervention plan) accompanied by continuous digital auditing remains essential to definitively isolate training-induced plastic alterations from baseline characteristics and to clarify any causal cognitive transfer to broader academic competencies, such as mathematical logic and executive processing skills.

## 5. Conclusions

In conclusion, this study demonstrates distinct, non-causal cross-sectional baseline differences in brain structures and intrinsic functional signaling indices between expert-level and novice-level chess-playing school-aged children. Expert-level proficiency co-varies with larger gray matter volumes, greater cortical thickness, and heightened spontaneous BOLD fluctuation indices spanning localized frontoparietal and limbic regions. Notably, the exploratory structural behavioral analysis revealed that the cortical thickness of the right subparietal sulcus exhibits a tight linear coupling with selected baseline 0-back VSWM accuracy. While these neurostructural and functional features overlap anatomically with core networks historically implicated in complex spatial processing, these cross-sectional findings capture purely associational alignments rather than demonstrate prospective training effects, and they do not support sweeping claims of generalizable educational benefits. Future prospective longitudinal intervention investigations accompanied by digital auditing remain imperative to definitively clarify any causal cognitive transfer to broader academic competencies.

## Figures and Tables

**Table 1:** Inclusion criteria.

Inclusion Criteria
School-aged children from basic education primary and secondary schools in Beijing; Visual acuity or corrected visual acuity greater than 0.8, with no color blindness or color weakness; Age 10–15 years; All participants required guardian consent to participate in this study; The MRIRC magnetic resonance safety screening checklist from the MRI Research Center, Institute of Psychology, Chinese Academy of Sciences (CAS), was used to confirm suitability for MRI scanning (e.g., no implanted metal such as metal dentures, no electronic, magnetic, or mechanical devices such as pacemakers); All participants and their guardians signed informed consent forms.

**Table 2:** Exclusion criteria.

Exclusion Criteria
Clear history of head trauma; Neurological or psychiatric disorders, such as epilepsy or tic disorders; Hearing or vision impairments; Short-term use of medications affecting the central nervous system; Students who had recently achieved a chess ranking or attended academic training classes; Students who had not participated in school courses for an extended period.

**Table 3:** Basic information of MRI participants in experimental and control groups.

	Experimental Group	Control Group
Number of participants (*n*)	9	11
Age (years)	13.32 ± 1.27	13.65 ± 1.19
Sex (M/F)	6/3	8/3
Chess rating (level)	Class 1 Knight	Understands chess knowledge and skills
Weekly study time (hours)	2.1 ± 0.16	None

**Table 4:** Basic information of behavioral study participants in experimental and control groups.

	Experimental Group	Control Group
Number of participants (*n*)	12	12
Age (years)	13.32 ± 1.27	13.65 ± 1.19
Sex (M/F)	7/5	9/3
Chess rating (level)	Class 1 Knight	Understands chess knowledge and skills
Weekly study time (hours)	2.1 ± 0.16	None

**Table 5:** Descriptive statistics for visual spatial working memory accuracy.

Visual Spatial Working Memory Task (N-Back) Accuracy	Experimental Group	Control Group	t	*p*	Cohen’s d
0-back	93.92 ± 2.07	86.29 ± 1.90	4.971	<0.001 ***	2.081
1-back	92.83 ± 2.65	92.50 ± 2.39	0.154	0.879	0.131

Note: *** *p* < 0.001.

**Table 6:** Descriptive statistics for visual spatial working memory reaction time.

Visual Spatial Working Memory Task (N-Back) Reaction Time	Experimental Group	Control Group	t	*p*	Cohen’s d
0-back	483.47 ± 34.37	518.81 ± 36.85	−2.438	0.023 *	−0.992
1-back	515.62 ± 41.67	533.07 ± 28.09	−1.190	0.246	−0.492

Note: * *p* < 0.05.

**Table 7:** Differences in brain gray matter volume between experimental and control groups.

Anatomical Location	Hemisphere	Experimental Group	Control Group	*p*	t
Superior occipital gyrus (SOG)	L	3.34	3.01	0.018 *	2.59
Precuneus (PCu)	R	11.80	10.67	0.032 *	2.30
Precuneus (PCu)	L	11.68	10.50	0.022 *	2.48
Supramarginal gyrus (SMG)	R	9.00	7.75	0.001 ***	4.43
Supramarginal gyrus (SMG)	L	9.30	8.39	0.017 *	2.61
Frontal operculum (FO)	L	2.06	1.82	0.017 *	2.62
Posterior cingulate cortex (PCC)	R	4.27	3.85	0.044 *	2.15
Cuneus	R	4.39	3.95	0.047 *	2.12
Planum polare (PP)	R	1.88	1.70	0.028 *	2.37
Angular gyrus (ANG)	L	3.34	3.01	0.040 *	2.20
Transverse temporal gyrus (TTG)	L	1.47	1.30	0.020 *	2.53
Triangular part of the inferior frontal gyrus (IFGtriang)	L	3.74	3.37	0.035 *	2.26

Note: Values represent the mean cluster gray matter volume expressed in milliliters (mL). Results from independent-sample *t*-test; * *p* < 0.05; *** *p* < 0.001.

**Table 8:** Non-significant brain regions for gray matter volume between experimental and control groups.

	Experimental Group (*n* = 9)		Control Group (*n* = 11)			
	M	SD	M	SD	t	*p*
Left superior frontal gyrus (L SFG)	37.071	2.467	35.053	2.788	1.721	0.101
Left middle frontal gyrus (L MFG)	29.369	2.107	27.656	3.024	1.451	0.163
Left postcentral gyrus (L poCG)	12.335	1.024	11.376	1.332	1.795	0.089
Right postcentral gyrus (R poCG)	11.553	0.785	10.872	0.952	1.745	0.097
Left superior parietal gyrus (L SPG)	14.695	1.580	13.645	1.245	1.707	0.104
Right superior parietal gyrus (R SPG)	14.517	1.476	13.633	1.185	1.524	0.144
Right angular gyrus (R ANG)	14.226	1.309	12.989	1.605	1.886	0.075
Right superior occipital gyrus (R SOG)	4.833	0.704	4.378	0.466	1.787	0.090

Note: Results from independent-sample *t*-test.

**Table 9:** Differences in brain cortical thickness between experimental and control groups.

Anatomical Location	Hemisphere	Experimental Group	Control Group	*p*	t
Triangular part of the inferior frontal gyrus (IFGtriang)	L	2.85	2.69	0.023 *	2.46
Angular gyrus (ANG)	L	2.74	2.61	0.029 *	2.36
Supramarginal gyrus (SMG)	L	2.88	2.75	0.021 *	2.50
Precuneus (PCu)	R	9.06	8.11	0.020 *	2.54
Transverse frontopolar gyri and sulcus (tFPG/s)	L	2.79	3.62	0.026 *	2.40
Middle frontal sulcus (mfs)	L	2.68	2.56	0.041 *	2.18
Sulcus intermedius primus of Jensen (si_Jensen)	L	2.77	2.55	0.010 **	2.88
Interparietal and transverse parietal sulcus (IPS/tPS)	L	2.46	2.35	0.020 *	2.54
Superior occipital and transverse occipital sulcus (SOS/tOS)	R	2.37	2.29	0.040 *	2.20
Postcentral sulcus (poCS)	R	2.44	2.31	0.042 *	2.17
Subparietal sulcus (sps)	L	2.66	2.56	0.014 *	2.70
Subparietal sulcus (sps)	R	2.69	2.50	0.008 **	2.94
Temporal pole (TP)	R	2.80	3.04	0.030 *	−2.34

Note: Results from independent-sample *t*-test; * *p* < 0.05; ** *p* < 0.01.

**Table 10:** Non-significant brain regions for cortical thickness between experimental and control groups.

	Experimental Group (*n* = 9)		Control Group (*n* = 11)			
	M	SD	M	SD	t	*p*
Left superior frontal gyrus (L SFG)	3.098	0.210	3.048	0.154	0.641	0.529
Left middle frontal gyrus (L MFG)	2.875	0.242	2.785	0.137	1.078	0.294
Right supramarginal gyrus (R SMG)	2.830	0.125	2.733	0.108	1.892	0.074
Left postcentral gyrus (L poCG)	2.231	0.157	2.160	0.134	1.116	0.278
Right postcentral gyrus (R poCG)	2.208	0.167	2.166	0.171	0.553	0.587
Left superior parietal gyrus (L SPG)	2.414	0.108	2.329	0.147	1.457	0.161
Right superior parietal gyrus (R SPG)	2.421	0.146	2.348	0.106	1.331	0.199
Right angular gyrus (R ANG)	2.693	0.153	2.685	0.120	0.128	0.899
Right superior occipital gyrus (R SOG)	2.286	0.098	2.220	0.119	1.353	0.192

Note: Results from independent-sample *t*-test.

**Table 11:** Differences in fALFF values between experimental and control groups.

Anatomical Location	Hemisphere	Peak Coordinates (x, y, z)	Voxel Size	t
Anterior cingulate cortex (ACC)	R	9, 36, 18	332	8.72 ***
Superior frontal gyrus, dorsolateral (SFGdl)	L	−15, 69, 3	293	6.60 ***
Orbitofrontal cortex (OFCant)	R	33, 42, −6	198	4.95 ***
Calcarine cortex (CAL)	L	0, −81, 6	147	−4.91 ***

Note: Results are from an independent-sample *t*-test; *** *p* < 0.01 and a cluster size (voxel) > 147 indicate significant differences.

## Data Availability

The datasets generated and analyzed during the current study are not publicly available due to ethical restrictions and institutional policies safeguarding the privacy of the child participants. Anonymized group-level statistical data or analytical derivatives may be made available from the corresponding author upon reasonable academic request for non-commercial research purposes.
